# Neuroligin-1 in brain and CSF of neurodegenerative disorders: investigation for synaptic biomarkers

**DOI:** 10.1186/s40478-021-01119-4

**Published:** 2021-02-01

**Authors:** Elena Camporesi, Tammaryn Lashley, Johan Gobom, Juan Lantero-Rodriguez, Oskar Hansson, Henrik Zetterberg, Kaj Blennow, Bruno Becker

**Affiliations:** 1 Department of Psychiatry and Neurochemistry, Institute of Neuroscience and Physiology, The Sahlgrenska Academy At University of Gothenburg, Mölndal, Sweden; 2 Clinical Neurochemistry Laboratory, Sahlgrenska University Hospital, Mölndal, Sweden; 3 The Queen Square Brain Bank for Neurological Disorders, Department of Clinical and Movement Neurosciences, UCL Institute of Neurology, London, UK; 4 Department of Neurodegenerative Disease, UCL Institute of Neurology, University College London, Queen Square, London, UK; 5 Clinical Memory Research Unit, Department of Clinical Sciences, Lund University, Lund, Sweden; 6 Memory Clinic, Skåne University Hospital, Malmö, Sweden; 7 Dementia Research Institute At UCL, London, UK; 8 Department of Neurodegenerative Disease, UCL Institute of Neurology, Queen Square, London, UK

**Keywords:** Neuroligin-1, Synapse loss, Alzheimer’s disease, Tauopathies

## Abstract

Synaptic pathology is a central event in Alzheimer’s disease (AD) and other neurodegenerative conditions, and investigation of synaptic proteins can provide valuable tools to follow synaptic dysfunction and loss in these diseases. Neuroligin-1 (Nlgn1) is a postsynaptic cell adhesion protein, important for synapse stabilization and formation. Nlgn1 has been connected to cognitive disorders, and specifically to AD, as target of the synaptotoxic effect of amyloid-β (Aβ) oligomers and Aβ fibrils. To address changes in Nlgn1 expression in human brain, brain regions in different neurological disorders were examined by Western blot and mass spectrometry. Brain specimens from AD (n = 23), progressive supranuclear palsy (PSP, n = 11), corticobasal degeneration (CBD, n = 10), and Pick’s disease (PiD, n = 9) were included. Additionally, cerebrospinal fluid (CSF) samples of AD patients (n = 43) and non-demented controls (n = 42) were analysed. We found decreased levels of Nlgn1 in temporal and parietal cortex (~ 50–60% reductions) in AD brains compared with controls. In frontal grey matter the reduction was not seen for AD patients; however, in the same region, marked reduction was found for PiD (~ 77%), CBD (~ 66%) and to a lesser extent for PSP (~ 43%), which could clearly separate these tauopathies from controls. The Nlgn1 level was reduced in CSF from AD patients compared to controls, but with considerable overlap. The dramatic reduction of Nlgn1 seen in the brain extracts of tauopathies warrants further investigation regarding the potential use of Nlgn1 as a biomarker for these neurodegenerative diseases.

**Supplementary Information:**

The online version of this article (10.1186/s40478-021-01119-4) contains supplementary material, which is available to authorized users.

## Introduction

Synapses are the neuron connecting units, essential for their communication, formed by a presynaptic compartment containing vesicles for storage and release of neurotransmitters, and a postsynaptic compartment containing receptors to capture and elaborate the signal and eventually, transmit it further [1]. Altered synaptic functions and loss of synapses underlie the cognitive impairment seen in Alzheimer´s disease (AD) [2–6], which is a progressive neurodegenerative brain disorder characterized by depositions of extracellular amyloid-beta (Aβ) plaques and intracellular accumulation of tau neurofibrillary tangles (NFTs) [7, 8]. Synapse loss is also observed in other neurodegenerative diseases [9, 10] and synaptic dysfunction has been reported in animal models of tauopathies [11, 12], a group of brain diseases aetiologically connected to tau dyshomeostasis [13] and different pathways linking tau to synaptic alterations have been described [14, 15]. AD neuropathological changes are detectable in the cerebrospinal fluid (CSF), where decreased levels of Aβ_42_ fragment (or the ratio Aβ_42/40_ [16]) reflecting amyloid pathology, increased levels of total-tau (*t*-tau) and phospho-tau (*p*-tau) protein, reflecting neuronal injury and tau pathology respectively, define an AD profile in living humans. These CSF biomarkers are part of the research diagnostic criteria for AD [17], although they have no diagnostic value for other neurodegenerative disorder. For instance, for the aforementioned tauopathies, no biomarkers are currently available.

Therefore, global efforts are made to screen for new CSF biomarkers with high diagnostic and prognostic values, being able to discriminate between different diseases and possibly detecting those at an early stage. Synaptic dysfunction appears to be an early event in AD [2, 18] and other neurodegenerative diseases, correlating with clinical symptoms, hence synaptic proteins can be candidates for early biomarkers. Many synaptic proteins have been investigated [19], however, more investigations are needed to be able to use these biomarkers as part of the diagnostic work-up of neurodegenerative diseases in the clinic. In addition, different synaptic proteins can reflect different pathological responses, therefore the investigation of many synaptic proteins can improve the understanding of the pathological processes affecting synapses during neurodegenerative diseases.

A proper synapse assembly is crucial for its correct functioning. Essential during synaptic formation and maintenance are synaptic adhesion proteins, among which neuroligins have been thoroughly characterized. Neuroligins are a family of type I transmembrane proteins expressed in humans by five genes [20, 21], with neuroligin-1, -2 and -3 being mainly expressed in the central nervous system (CNS) [22]. Neuroligin-1 (Nlgn1) is specifically localized at the postsynaptic compartment of excitatory synapses [23], where it binds to its presynaptic partner neurexin-1β, stabilizing the synapse [24, 25]. Nlgn1 is important for synapse development and function [26], and both its extracellular part and the C-terminal domain seem to have distinct, but equally necessary, roles [27–31]. Nlgn1 extracellular domain undergoes proteolytic cleavage near to its transmembrane domain [32, 33] leading to the release of a soluble extracellular fragment. This ectodomain shedding appears to be activity-dependent, thus linking Nlgn1 to regulation of synaptic transmission in development and plasticity [32–34]. Nlgn1 has been shown to be necessary for long-term potentiation (LTP) [30, 35, 36], one of the best-known form of synaptic plasticity, and neuroligin-knockout mouse models show a decrease in LTP [37], while overexpression of the protein leads to an increased number of glutamatergic synapses as well as increased synaptic activity [25, 38]. Although the exact mechanisms by which Nlgn1 regulates synaptic structure and function are not yet well understood, the numerous studies that are aimed at unravelling those mechanisms underline the importance of this synaptic protein in synapse homeostasis.

Nlgn1 has been connected to cognitive disorders [39], while others have investigated specifically the possible involvement of Nlgn1 in AD and neurodegenerative conditions [40–42]. Nlgn1 has been described, both in vivo and in vitro, as a possible target for Aβ oligomers (Aβo), which are considered to induce synaptotoxicity in AD [43, 44]. Additionally, a recent study [45] showed a reduction of the protein in hippocampus of AD patients, which was modulated by Aβ load. Taken together, these studies suggest that Nlgn1 protein levels might be altered during AD and possibly connected to Aβ-related neurodegenerative effects.

In this exploratory study, we aimed to test the hypothesis that Nlgn1 protein levels change in the brain during neurodegenerative disorders, possibly yielding CSF biomarkers for those diseases. We initially focused on AD, but then expanded the study of Nlgn1 changes in brain to a group of primary tauopathies to be able to investigate Nlgn1 in relation to both Aβ and tau. Multiple brain regions of AD patients as well as from patients with corticobasal degeneration (CBD), progressive supranuclear palsy (PSP) and Pick’s disease (PiD) were analysed. Finally, we aimed at verifying the presence of the protein or its fragments in CSF of AD cases versus controls.

## Materials

### Post-mortem human brain tissue

Human brain tissue samples were obtained from Queen Square Brain Bank for Neurological Disorders (QSBB), Department of Clinical and Movement Neurosciences, Institute of Neurology, University College London (UCL) and Netherlands Brain Bank and were stored at − 80 °C pending homogenization and biochemical analysis. Samples description and case demographics are shown in Table 1 (full demographic data shown in Additional file 1: Table S1, Additional file 2: Table S2). Human brain tissues were used in accordance with the Helsinki declaration and the regional ethics committees at UCL and the University of Gothenburg.

**Table 1:** Demographics and clinical characteristics of subjects included in study 1, 2 and 3

	Control	sAD	fAD	PA
Study 1	n = 4	n = 4		
Brain region	Parietal cortex	Parietal cortex		
Gender (male/female)	1/3	1/3		
Age at death (years)	64.25 [3.40]	64.50 [2.52]		
Post-mortem delay, (h)	6.66 [1.19]	5.72 [0.59]		
Braak stage 0-I/II-IV/V-VI	4/0/0	0/0/4		
Nlgn1 band signal (F-7 ab)	4425 [419.5]	2374 [914.5]		

	Control	sAD	fAD	PA
Study 2	n = 7	n = 9	n = 9	n = 15
Brain region	Temporal cortex	Temporal cortex	Temporal cortex	Temporal cortex
Gender (male/female)	4/3	6/3	3/6	4/11
Age at death (years)	74.14 [18.42]	72.11 [8.3]	52.67 [9.99]	86.33 [5.96]
Post-mortem delay (h)	58.96 [22.77]	71.17 [19.05]	38.8 [17.74]	76.25 [31.08]
Braak stages 0/I-II/III-IV/V-VI	5/2/0/0	0/0/0/9	0/0/0/9	0/4/11/0
Thal phases 0/1–2/3/4–5	5/2/0/0	0/0/0/9	0/0/0/9	0/4/5/6
CERAD score (0/1/2/3)	6/1/0/0	0/0/1/8	0/0/0/9	2/8/5/0
CAA (0/1/2/3)	5/1/1/0	0/4/3/2	0/1/1/7	6/2/6/1
Nlgn1 WB quantification (samples ng/QC ng; 20 µg brain homogenate)	2.22 [0.96]	0.85 [0.57]	0.97 [0.58]	1.78 [0.80]

	Control	sAD	PSP	CBD	PiD
Study 3	n = 10	n = 10	n = 11	n = 10	n = 9
Brain region	Frontal grey matter	Frontal grey matter	Frontal grey matter	Frontal grey matter	Frontal grey matter
Gender (male/female)	4/6	6/4	6/5	7/3	8/1
Age at death (years)	78.7 [17.65]	69.4 [8.13]	79.27 [9.04]	68.6 [4.93]	69.78 [4.79]
Post-mortem delay, (h)	77.45 [42.8]	56.05 [26.32]	47.93 [21.63]	72.86 [25.05]	67.39 [27.28]
Braak stages 0/I-II/III-IV/V-VI	3/7/0/0	0/0/0/10	na	na	na
Thal phases 0/1–2/3/4–5	4/4/1/1	0/0/0/10	na	na	na
CAA (0/1/2/3)	8/2/0/0	1/1/1/7	na	na	na
Nlgn1 WB quantification (samples ng/QC ng; 20 µg brain homogenate)	0.92 [0.29]	0.56 [0.35]	0.65 [0.33]	0.45 [0.21]	0.42 [0.27]

The data are presented as mean and [standard deviation]

*WB * Western blot, *Na * not available, *CAA* cerebral amyloid angiopathy, 0 = none, 1 = mild, 2 = moderate, 3 = severe

**Table 2:** Demographics and biomarkers of CSF samples used in study 4

Study 4	Controls (n = 42)	AD (n = 43)
Gender (male/female)	1/3	1/3
Age (years)	72.64 [6.29]	74.17 [7.71]
Clinical group, MMSE*	29.24 [0.76]	20.05 [4.31]
AD Biomarkers		
Aβ_40_ [pg/ml]	6690 [2139]	6523 [1887]
Aβ_42_ [pg/ml]	770.6 [303.2]	409.6 [130.2]
*t*-Tau [pg/ml]	365.3 [193.4]	619.3 [200]
*p*-Tau [pg/ml]	50.81 [35.26]	116.4 [42.97]
Nlgn1signal ratio (F-7 ab)	0.22 [0.11]	0.17 [0.13]

* Based on the Mini-Mental State Examination (MMSE) score, a score of 20 to 24 suggests mild cognitive impairment (MCI), 13 to 20 suggests moderate dementia and less than 12 indicates severe dementia

All the data are presented as mean and [standard deviation]

#### Individuals included in study 1

Brain tissue samples from the superior parietal gyrus were obtained from the Netherlands Brain Bank. Samples from AD patients fulfilled the criteria for Braak stages V or VI and the controls for Braak stage 0, in accordance with the Braak and Braak criteria [46].

#### Individuals included in study 2: AD cohort

Human post-mortem brain tissue from temporal cortex were obtained through the brain donation program at QSBB. This AD cohort was composed of sporadic AD (sAD; n = 9), familial AD cases (fAD; n = 9), controls (Ctrl; n = 7) and pathological aging (PA; n = 15). The term PA was used to refer to individuals with AD neuropathological changes at autopsy without having any history of cognitive impairment at the time of death. These cases cannot be clinically classified as AD, because of non-apparent antemortem cognitive decline, neither as controls because of the significant high presence of AD-like pathology. It is not yet clear why these individuals respond differently to neuropathological changes, but because of these characteristics, they have been suggested to represent a preclinical stage in the Alzheimer´s continuum [47]. Based on published consensus criteria [48], all autopsies included tissue sampling in regions relevant to the differential diagnosis of dementia. AD patients met the 2012 NIA-AA guidelines for neuropathologic assessment [49] and fulfilled the clinical NINCDS criteria for probable AD [50]. Thal phases, describing the spreading of Aβ pathology (phase 0-no amyloid; phase 1–2-isocortical; phase 2-limbic; phase 3-basal ganglia; phase 4-basal forebrain and midbrain; and phase 5-pons/medulla oblongata and cerebellum); Braak and Braak staging, corresponding to the spread of tau pathology (NFTs) (stage I-transentorhinal, stage III-IV-limbic; stage V-VI-isocortical); CERAD scoring system assessing the frequency of the neuritic plaques deposition (0-none, 2-sparse, 3-moderate, 4-frequent [51]), were determined according to the corresponding criteria [46, 52, 53]. Cerebral amyloid angiopathy was assessed using a three tier scoring system [54].

#### Individuals included in study 3: tauopathies cohort

Human post-mortem brain tissues from frontal grey matter was obtained through the brain donation program at QSBB. In this cohort, n = 10 sAD, n = 10 Ctrl, n = 11 PSP, n = 10 CBD and n = 9 PiD samples were included. Neuropathological diagnosis was made according to diagnostic criteria for AD [49], PSP, CBD and PiD [55]. These diseases are recognized as frontotemporal lobar degeneration [56]. For comparison between diseases the frontal cortex was used as the affected area.

### Human CSF sample subjects used in Western blot study 4

CSF samples were obtained through our collaboration with Lund University and are part of the Swedish BioFINDER study (http://biofinder.se/). CSF samples were collected by lumbar puncture procedure at the Memory Clinic and Clinical Memory Research Unit in Malmö, Sweden. In the study, clinically diagnosed AD patients (n = 43) and healthy controls (n = 42) were analysed. One individual from the AD group lacked CSF AD biomarker results. Mini-Mental State Examination (MMSE) was used to measure cognitive impairment. The study was conducted according to the Helsinki Declaration and approved by the regional ethical board in Lund (Dnr 695/2008). Demographics and biomarker characteristics of the patients are shown in Table 2.

## Methods/experimental procedures

### Homogenization of brain tissues for Western blot analysis

Approximately 100 mg (± 20 mg) of frozen brain tissues were manually cut and 1 mL TBS buffer (20 mM Tris–HCl, 137 mM NaCl, pH = 7.6, with Complete Protease inhibitor Cocktail, Roche Diagnostic GmbH) was added. All the steps were performed on ice. Samples were homogenized for 2 min at 200 Hz using the Tissue Lyser II (Qiagen). After homogenization, samples were centrifuged for 1 h at 31,000 × *g* at + 4 °C and the supernatant (representing the soluble fraction) was aliquoted and stored frozen at − 80 °C. The same protocol was used for all brain samples. The protein concentration of the various TBS extracts was determined using the DC Protein Assay kit (Bio-Rad).

### Western blot analysis

TBS brain samples (20 µg) were mixed with XT sample buffer containing lithium dodecyl sulphate (LDS) and XT reducing agent (Bio-Rad Laboratories, cat# 1,610,791 and cat# 161–0792, respectively). For the CSF study, samples of 17.5 µL of neat CSF were used and prepared in the same way. Samples were heated for 10 min at 70 °C and subsequently loaded onto 4–12% Criterion™ XT Bis–Tris gels, which were run using XT MES buffer (Bio-Rad Laboratories, cat# 1,610,789). On each gel, recombinant human Nlgn1 fusion protein (R&D System cat# 7066-NL), corresponding to the extracellular part of the protein, was added to generate a calibration curve for quantification. Nlgn2 and Nlgn3 recombinant proteins were also used for antibody specificity checking (R&D System cat# 5645-NL-050 and cat# 9069-NL-050 respectively). Additionally, same amounts of a control brain sample (QC) were included in every blot to correct for inter-gel variations. SeeBlue Plus2™ (Thermofisher) pre-stained protein standard was used as molecular weight marker. Gels were subsequently equilibrated in transfer buffer (NuPAGE™; ThermoFisher NP0006) containing 20% v/v methanol for 25 min and then transferred to a 0.2 µm nitrocellulose membrane (Amersham cat# 10,600,001) using a semi-dry blotting apparatus for 1 h (constant current, approx. 0.7 mA/cm^2^). Membranes were blocked using 5% non-fat dry milk (Bio-Rad Laboratories, cat# 1,706,404) in PBST (PBS, with added 0.05% Tween 20, BioRad Laboratories, cat #1,610,781) for 1 h. Thereafter, membranes were incubated overnight with anti-Nlgn1 primary antibodies (sc-365087, F-7 Santa Cruz Biotechnology, concentration 0.4 µg/mL; a-Nlgn1 Synaptic Systems, cat# 129,111, concentration 1 µg/mL) or no primary antibody (negative control) at + 4 °C. Membranes were washed 3 × 10 min with PBS-Tween before incubation with anti-mouse IgG specific secondary antibody (Santa Cruz Biotechnology, sc-516102) HRP conjugated for 1 h at room temperature, diluted 1:5000. Primary and secondary antibodies were diluted in PBST with added 0.1% bovine serum albumin (BSA, Sigma-Aldrich). After 3 × washing for each 10 min with PBST, membranes were developed with ECL Select™ Western Blotting Detection Reagent (GE Healthcare) for 2 min, imaged on a Fujifilm LAS-3000 System (FUJIFILM Corporation) imager and quantified using ImageJ software (Rasband, WS, ImageJ; NIH, Bethesda, MD, http://rsb.info.nih.gov/ij). All the Western blots of human brain extracts were subsequently incubated with GAPDH antibody (cat# NB300-328H, Novus biological, 1 mg/mL; used at dilution 1:30,000) for 1 h at room temperature and developed in the same way. The positions of the samples corresponding to different diseases were randomized across the gels. Nlgn1 protein amounts in the samples were determined using the calibration curve obtained by a linear-curve fitting model. Brain samples were normalized against GAPDH signal, used as control for equal loading. All the samples (brain and CSF) were thawed and prepared the same day of the Western blot analysis.

### In-gel digestion and mass spectrometric analysis

Two µg of recombinant Nlgn1 protein, 30 µg of brain extract sample and 19.6 µL of CSF were analysed by SDS–polyacrylamide gel electrophoresis under reducing conditions as described before. Upon electrophoresis, proteins were visualized by SimplyBlue™ SafeStain Coomassie (ThermoFisher Scientific, cat# LC6060). Using the recombinant Nlgn1 protein band as a reference, bands of interest were excised from the brai n extract and CSF lanes, cut into pieces of ≈1 mm^3^ and destained using a 1:1 mixture of acetonitrile and 50 mM ammonium bicarbonate solution. Gel pieces were further washed and dried using a vacuum centrifuge. Subsequently, the gel pieces were reduced with 10 mM dithiothreitol (DTT) at 56 °C for 1 h and alkylated with 55 mM iodoacetamide for 45 min in the dark. Samples were digested over night with 10 ng/μl trypsin (Sequencing Grade Modified Trypsin, Cat# V5111 Promega) at 37 °C. The next day, digestion was stopped by the addition of 2% trifluoroacetic acid, 75% acetonitrile solution. The solution (containing the peptides) was transferred to a new collection tube. Gel pieces were further extracted with the addition of 50% acetonitrile, 0.2% trifluoroacetic acid solution for 30 min and the supernatant was transferred to the collection tube. Pooled extracts for each gel band sample were dried through vacuum centrifugation and dissolved in 42 μl 0.1% BSA, 0.05% trifluoroacetic acid solution. Forty μl per sample were analysed by LC–MS, using a nano-flow (300 nl/min) HPLC (Dionex 3000 system, Thermo Scientific), configured in a trap-column configuration, interfaced with a high resolution mass spectrometer (Orbitrap Fusion™ Tribrid™, Thermo) via an Easy-Spray™ ion source (Thermo Scientific). Mass spectrometric data was recorded in the data-dependent mode. Processing of MS data was performed using the software Proteome Discoverer 2.2 (Thermo). Protein identification was performed using the software Mascot (v.2.6.1, Matrixscience, UK). See also supplementary data.

### CSF analysis of AD core biomarkers

In the CSF study, EUROIMMUN assays were utilized to measure AD CSF biomarkers Aβ_40_ (#EQ 6511–9601-L), Aβ_42_ (#EQ 6521–9601-L), *t*-tau (#EQ 6531–9601-L) and *p*-tau (#EQ 6591–9601-L) according to the instructions of the manufacturer. Samples were dichotomized in Aβ + /Aβ- groups, using the Aβ_42/40_ ratio, as explained below. Further, the cut-off values for *t*-tau and *p*-tau were applied, to obtain a biologically defined AD group.

### Statistical analysis

Statistical analysis was performed using GraphPad Prism 7 (GraphPad Software, La Jolla, USA) and IBM SPSS, version 26. Using Shapiro–Wilk test, data were found to be non-normally distributed, thus non-parametric tests were applied. Differences between groups were assessed using Kruskal–Wallis test, followed by Dunn’s post-hoc test for multiple comparison, or Mann–Whitney t-test when appropriate. All the tests were two-sided and a *p* ≤ 0.05 was regarded as the threshold for significance. Data were adjusted by age, sex, and in case of the brain samples, post-mortem delay and brain weight also. Correlations were assessed using Spearman’s rank correlation. Additionally, in Study 2 associations between Nlgn1 and Aβ and tau pathology were assessed by plotting the Western blot values of Nlgn1 according to both, Braak, Thal and CERAD staging. Group differences between Nlgn1 values and combined Braak stages (0, I-II, III-IV, V-VI) and between Nlgn1 and combined Thal phases (0, 1–2, 3, 4–5) were tested. In the CSF analysis, the cut-offs for Aβ_42/40_ ratio, *t*-tau and *p*-tau were defined as the intercept between the two normal distributions resulting from a bimodal analysis using the *mixtools* package [57] in the statistical software R, as previously described [58].

## Results

### Use of two different antibodies for Nlgn1 detection identified the same reactive band in human brain samples

**Fig. 1:**
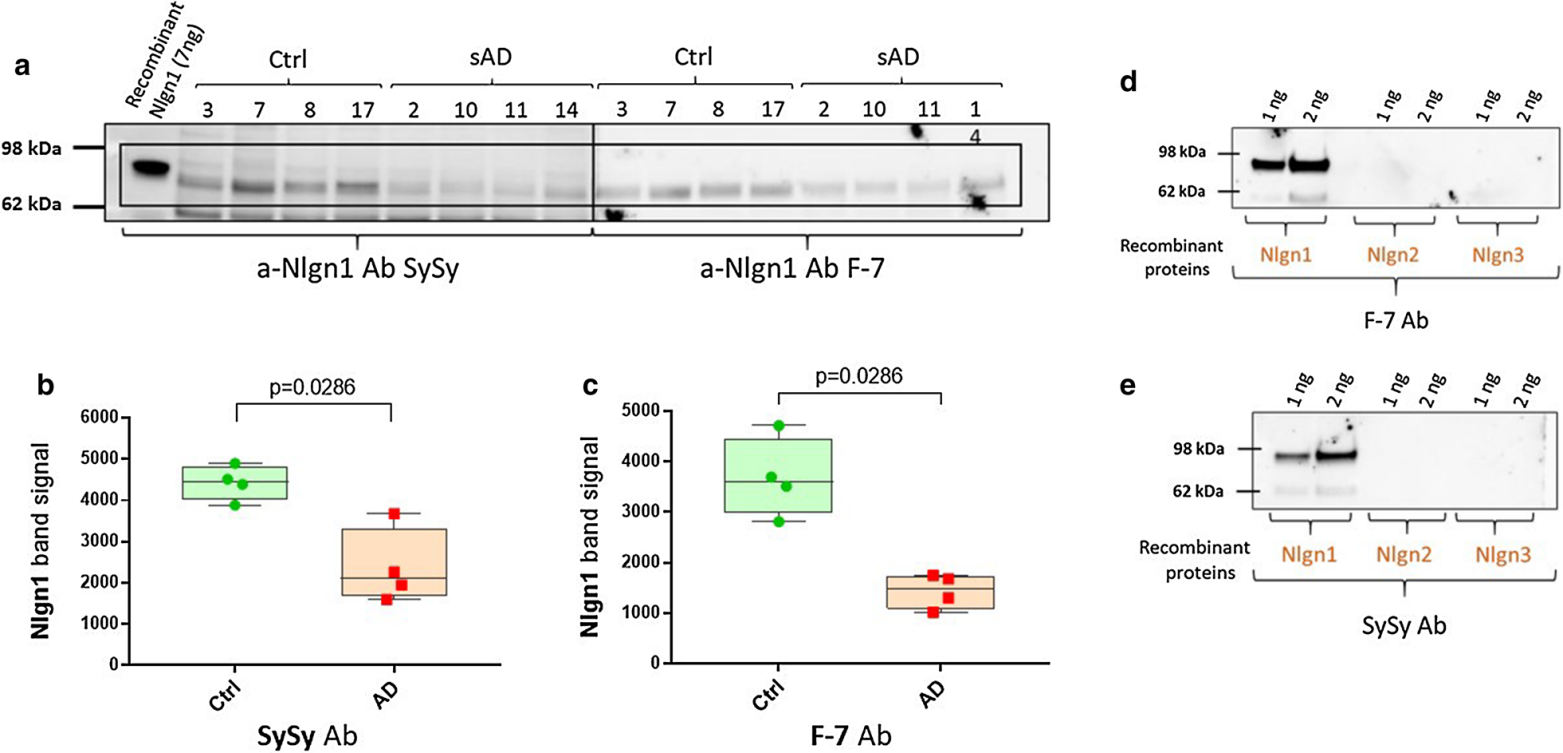
Reduction of Nlgn1 band in parietal cortex of AD brain. **a** Detection of Nlgn1 with antibodies targeting different epitopes by immunoblot. TBS soluble fractions of 4 Ctrl and 4 AD brain samples, 20 µg protein/lane. Samples 3–8–17–2–10–11-14 are from superior parietal cortex, while sample 7 is from inferior parietal cortex. **a** The blue-boxed areas indicate the common Nlgn1 immunoreactive band at around 80 kDa. Recombinant Nlgn1 protein, corresponding to the extracellular part fused to a C-terminal 6-His tag, is shown in the first lane. **b** Significant reduction of Nlgn1 band intensity, detected by a-Nlgn1 antibody from Synaptic System. **c** Significant reduction of Nlgn1 band intensity, detected by a-Nlgn1 (F-7) antibody. The bars presented in the scatterplots show the median and the interquartile range. Significance: *p*  ≤ 0.05. **d**–**e** Western blots showing the specificity of F-7 and SySy a-Nlgn1 antibodies toward Nlgn1 recombinant protein. Ab = antibody

**Fig. 2:**
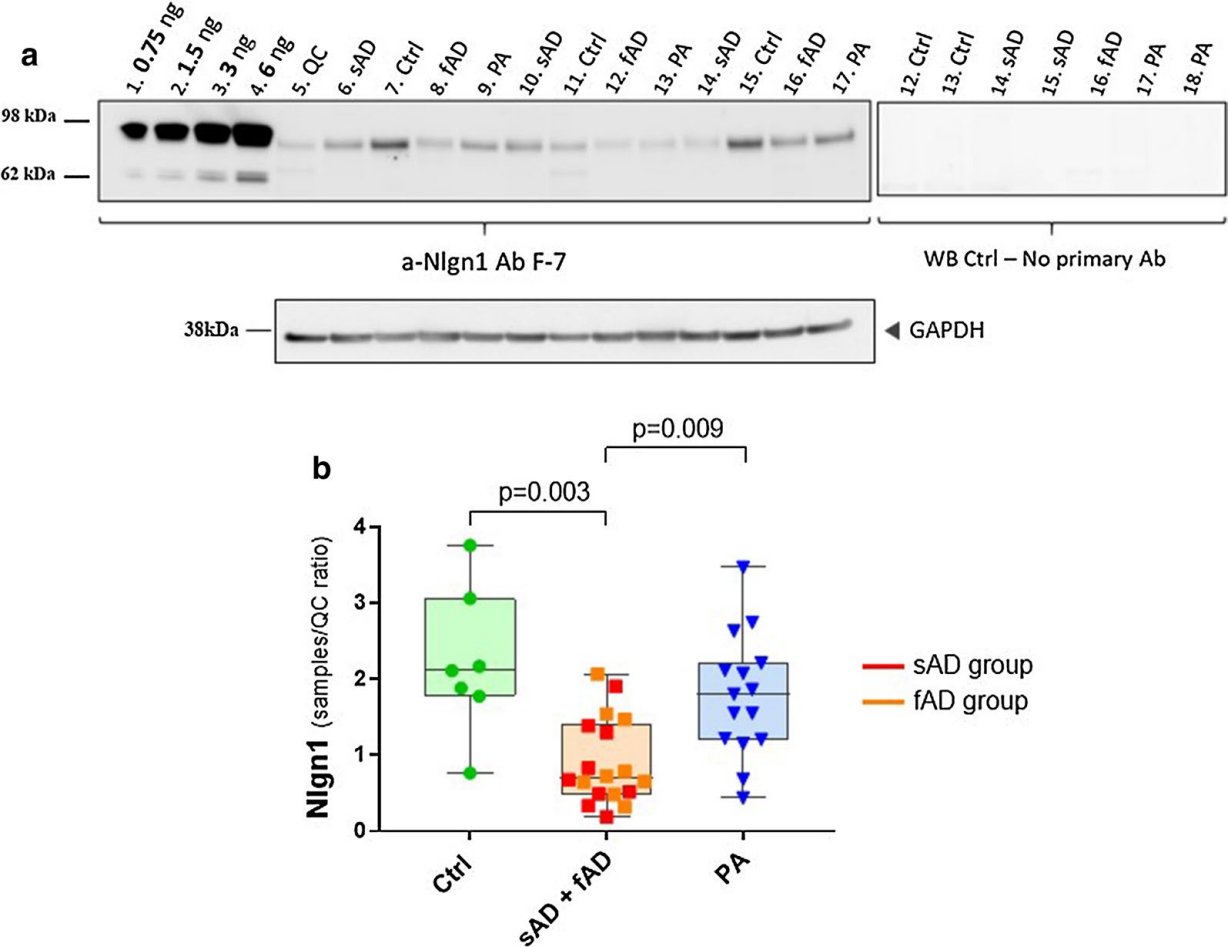
Reduction of Nlgn1 levels in temporal cortex of sporadic and familial AD cases. **a** Representative Western blot and relative GAPDH signal. Fifteen µg proteins per well of TBS soluble fractions of patients with sAD (n = 9), fAD (n = 9), PA (n = 15) and healthy Ctrl (n = 7) were separated by SDS-PAGE (4–12%) under reducing conditions. Neuropathological classification was based on Thal phases, Braak staging and CERAD score. A calibration curve was created using recombinant Nlgn1 extracellular domain fusion protein, to estimate the amount of Nlgn1 present in the samples. Samples were run on duplicate blots. **b** The box plots present the median and the interquartile range, the whiskers show the minimum and the maximum values. Significance: *p* ≤ 0.05. WB = Western blot

Nlgn1 has a molecular mass of approximately 96 kDa and contains a large extracellular domain, which undergoes shedding of the extracellular domain [32]. To test whether western blots could be used to detect Nlgn1 shedding in AD, TBS-soluble brain extracts were used and analysed with two different antibodies targeting the extracellular domain of the protein. Parietal cortex from four control and four AD brains were analysed. The two monoclonal antibodies, anti-Nlgn1 (SySy) and F-7, were able to identify the same band with an apparent molecular weight of 80 kDa. To check for Nlgn1 antibody specificity, both of the antibodies were probed against different concentrations of Nlgn1, Nlgn2 and Nlgn3 recombinant proteins. For both antibodies, a positive reactive band was observed only with Nlgn1, confirming no cross-reactivity with other neuroligins (Fig. 1). The intensity of the Nlgn1 band appeared to be decreased in the AD group as compared to the Ctrl group (*p* = 0.03) (Fig. 1). In this pilot study, where the emphasis was on the qualitative detection of Nlgn1, equal amounts of proteins were used for analysis. In the subsequent studies, (Figs. 2 and 4) normalisation by the housekeeping protein GAPDH was applied.

### Analysis of Nlgn1 levels showed a decrease in temporal cortex of human brain samples of AD patients

To validate these initial findings, a larger set of samples of TBS brain extracts from a second cohort, consisting of subjects with sAD, fAD, PA, and healthy controls, was analysed by western blot (Fig. 2).

Recombinant Nlgn1 fusion protein was used to generate a calibration curve for quantitation of Nlgn1 protein present in the samples. The analysis revealed significantly decreased levels of Nlgn1 protein in the AD group (sporadic and familial cases) compared to controls (*p* = 0.003) and the PA group (*p* = 0.009) (Fig. 2). No differences were observed between PA and controls, nor between sAD and fAD. Age, sex, brain weight and post-mortem delay were investigated as covariance. Sex, brain weight and post-mortem delay did not differ between the groups and did not influence the results when correcting for them. Regarding age differences, familial AD cases significantly differed in age when compared to the other groups, showing a younger age at death. The PA group was the oldest, showing a significantly higher age at death than sAD (*p* = 0.027), but it did not differ from the control group. When checking for correlations between age and Nlgn1 levels in individual groups, only the familial cases showed decreasing levels of Nlgn1 with age (rho = − 0.617). Despite having significant differences (*p* < 0.001) in group age between sAD and fAD, this was not reflected by different levels of Nlgn1 among these two groups, both having low levels of Nlgn1, not significantly different from each other. These evidences indicate that age did not have an effect on the low Nlgn1 protein level in the sAD and the fAD groups.

**Fig. 3:**
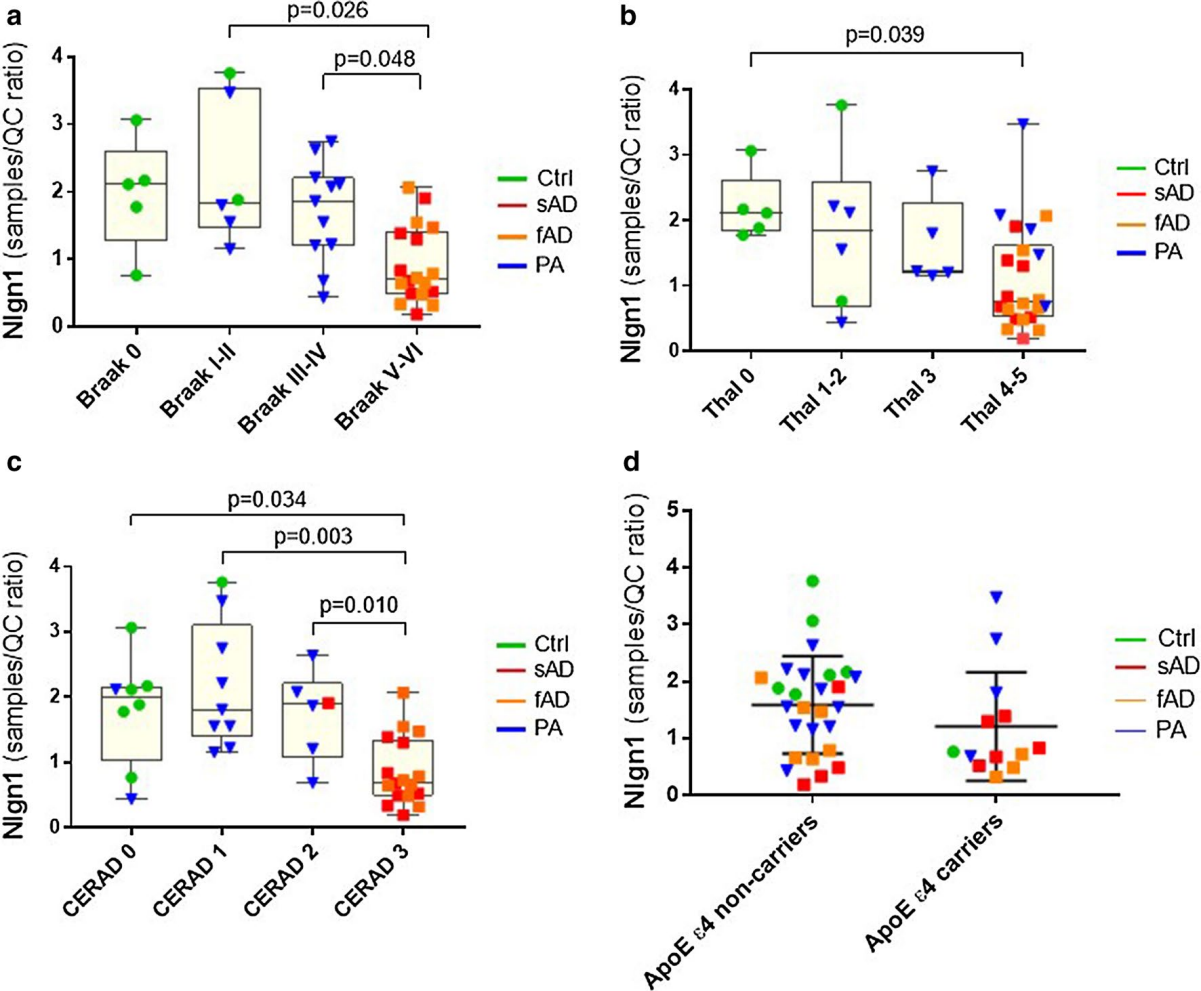
Nlgn1 level changes in relation to pathological classification. **a** Nlgn1 levels (as presented in Fig. 2) categorized by Braak stages (Braak 0, NFTs score 0, Braak I-II, transentorhinal; Braak III-IV, limbic; Braak V-VI, isocortical) **b** Nlgn1 levels classified based on Thal phases (Thal 0 = no amyloid; Thal 1–2 = isocortical; Thal 3 = basal ganglia; Thal 4 = forebrain and midbrain; Thal 5 = medulla oblongata and cerebellum). **c** Nlgn1 levels categorized by CERAD, neuritic plaques score (0 = none, 1 = sparse, 2 = moderate, 3 = frequent). **d** Nlgn1 levels categorized by ApoE allele ε4 carriers. In the box plots, median and interquartile range are represented by the box; whiskers represent the minimum and maximum values. In graph **d**, horizontal lines in the scatterplot represent median and interquartile range. Significance: *p*  ≤ 0.05

The groups were then investigated in relation to the pathological diagnosis and classified as a function of Braak stages, Thal phases and CERAD scores (Fig. 3). Classification according to Braak stages (scoring for the spread of tau NFTs) showed lower level of Nlgn1 across stages, with Braak V-VI showing significantly lower levels as compared to Braak I-II (*p* = 0.026) and Braak III-IV (*p* = 0.048) (Fig. 3a). Nlgn1 levels did not show a significant difference across Thal phases, except between Thal phase 0 and Thal phase 4–5, when high Aβ deposition was present (*p* = 0.039) (Fig. 3b). The same trend as observed for the Braak stages was seen in relation to CERAD score, where Nlgn1 levels were significantly lower with increased neuritic plaques score (Fig. 3c). Nlgn1 level did not show any difference in relation to CAA pathology or in relation to the *APOE* ε4 allele (Fig. 3d).

### The Nlgn1 level in frontal grey matter is strongly decreased in tauopathies

**Fig. 4:**
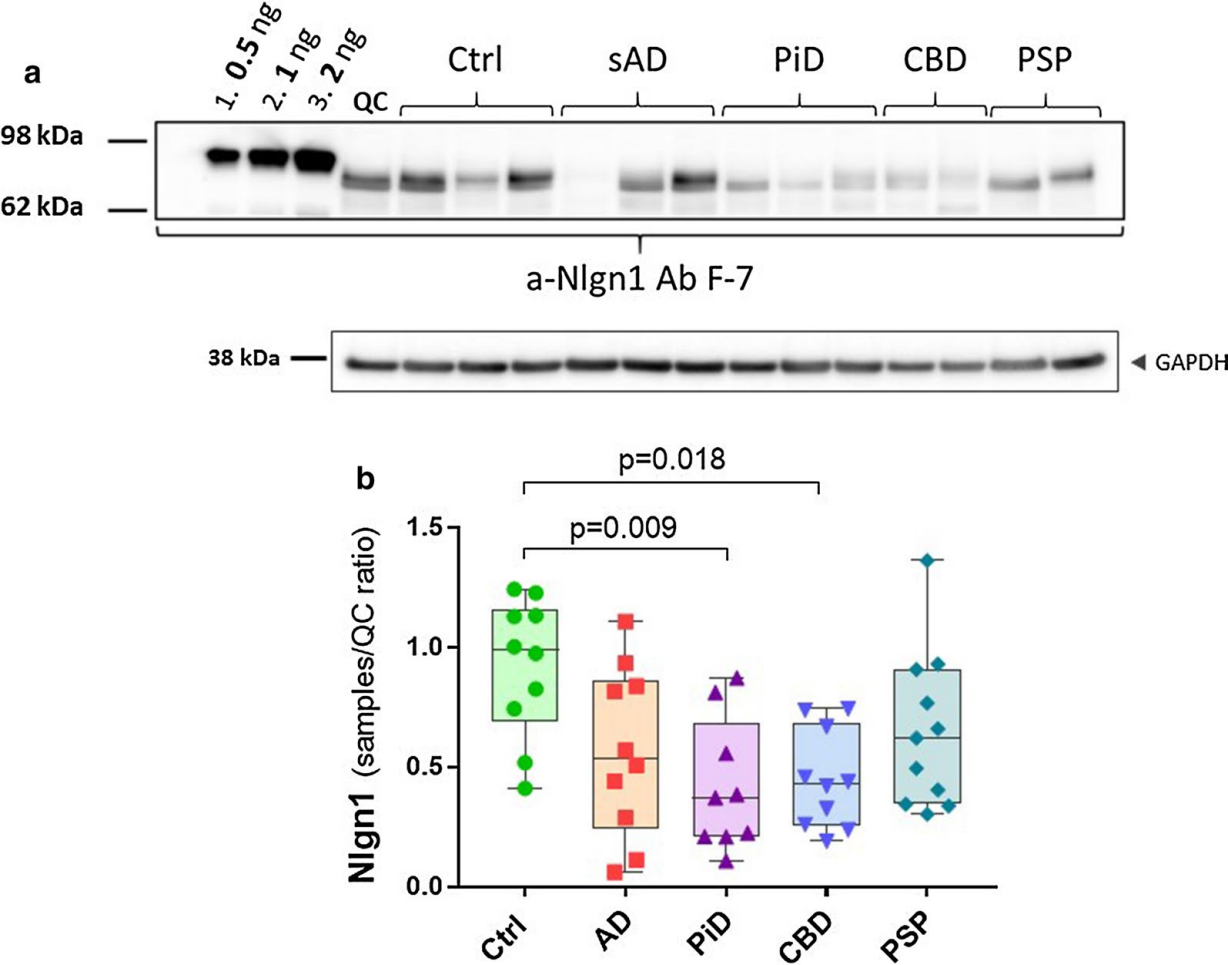
Nlgn1 reduction in frontal grey matter of tauopathies. Nlgn1 levels analysed in a tauopathies cohort including AD (n = 10), PiD (n = 9), CBD (n = 10), PSP (n = 11) and Ctrl (n = 10) from frontal grey matter. **a** representative Western blot and relative GAPDH signal. Samples were run as singlicates. A calibration curve of recombinant Nlgn1 fusion protein was used to estimate the amount of Nlgn1 present in the samples (as above). **b** The bars presented in the scatterplots show the median and the interquartile range. Significance: *p* ≤ 0.05

In order to explore the relation between Nlgn1 and tau deposition, we analysed samples from frontal grey matter of pathologically diagnosed PSP, CBD and PiD. In this study, sAD and control groups from the same brain area were also included. Data were corrected for age, sex and post mortem delay and none of these confounder variables significantly influenced the outcome. In CBD and PiD groups, Nlgn1 levels were significantly lower than control (*p* = 0.009 and *p* = 0.018, respectively), but no difference was found in the PSP group, even though a reduction trend was present. In this sample set from frontal brain areas, there were no significant differences between sAD and control or between sAD and the other tauopathies (Fig. 4).

### Nlgn1 is detectable in CSF and the protein levels are decreased in AD patients

**Fig. 5:**
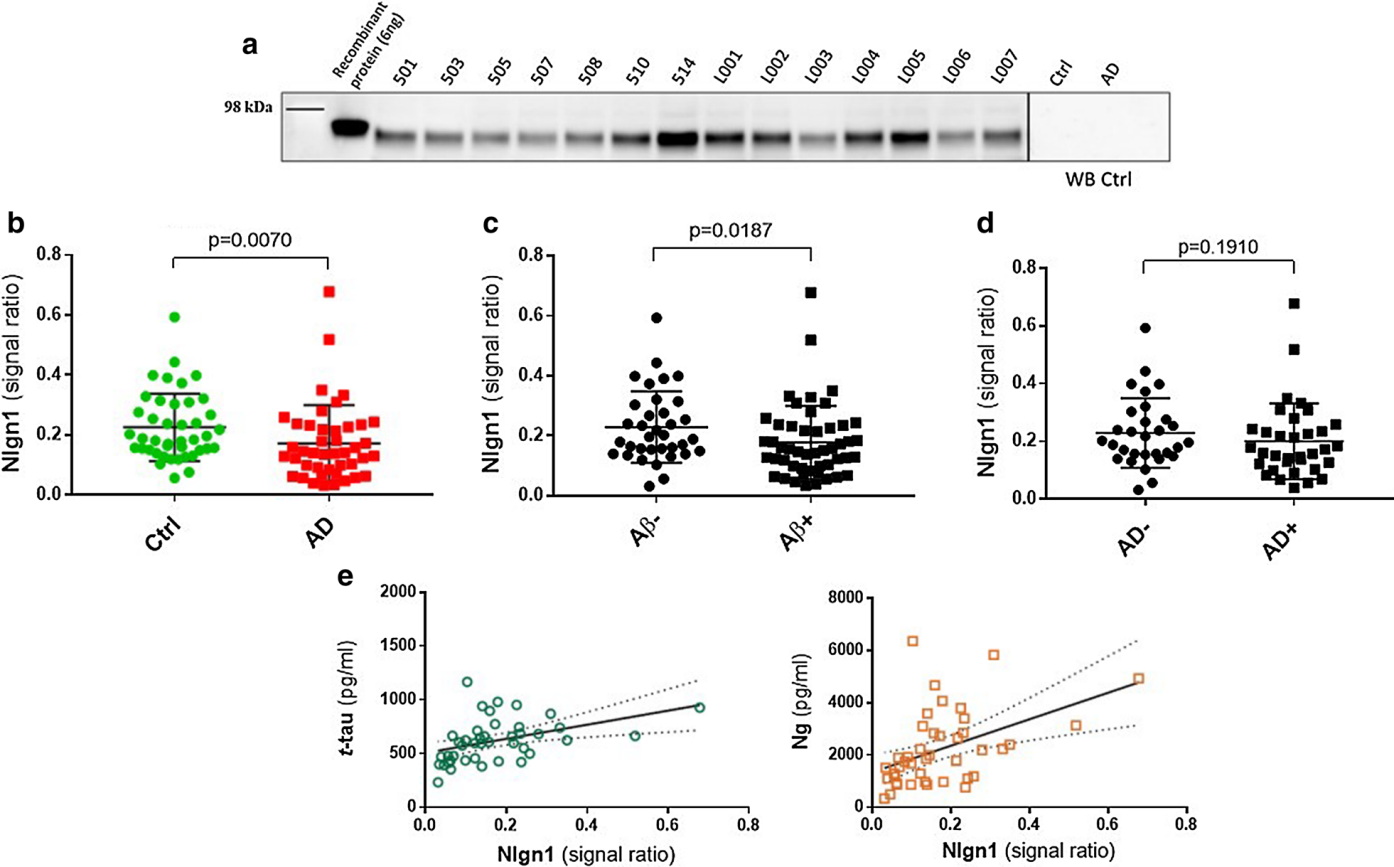
Nlgn1 levels do not differ in CSF of AD patients as compared to controls. Western blot analysis of Nlgn1 levels in CSF samples of AD (n = 43) and Ctrl (n = 42) individuals. Nlgn1 band was normalized using recombinant Nlgn1 to correct for inter-gel variation. **a** Representative Western blot. **b** Clinically characterized Ctrl and AD group **c** dichotomized Aβ_42*/*40_–negative (Aβ-) and Aβ_42*/*40_–positive (Aβ +) groups **d** AD biologically defined groups based on *t*-tau (cut-off = 460 pg/ml), *p*-tau (cut-off = 58.3 pg/ml) and Aβ_42*/*40_ (cut-off = 0.0934 pg/ml). The horizontal lines represent the median and interquartile range. Significance: *p* ≤ 0.05. **e** Correlation between CSF Nlgn1 levels and *t*-tau (left, Spearman´s rho = 0.547) and neurogranin (Ng) (right, Spearman´s rho = 0.512). Dashed lines represent the 95% confidence bands of the best-fit line

In order to investigate Nlgn1 after shedding from the postsynaptic membrane, we analysed CSF samples from clinically diagnosed AD patients (n = 43) and Ctrl subjects (n = 42) by Western blot using the same antibodies as in the study of brain homogenates (Fig. 5). CSF samples were sorted in groups classified according to clinical features based on MMSE score. Patients with low median MMSE score of 20 where classified as AD, while patients having a scores of 29 or above, were classified as controls (see Table 2). Bands of the same apparent molecular weight as in brain samples were detected. Recombinant Nlgn1 fusion protein was used as a QC sample on all blots to normalize the signals originating from several individual blots. The analysis (Fig. 5a) showed a reduction of Nlgn1 band intensity in the AD group as compared to control (*p* = 0.007). The MMSE test, together with other neuropsychological tests, is a very useful tool to assess the patient’s cognitive status, but because of comorbidities and overlap between symptoms a definitive diagnosis is challenging [59]. Therefore, according to the new biological definition of AD [17], we divided the samples based on Aβ_42/40_ ratio, *t*-tau and *p-*tau cut-off values, to get a disease-linked biochemical classification, not only based on the patient’s cognitive abilities. The groups were dichotomized based on the Aβ_42/40_ ratio (cut-off = 0.0934 pg/ml), with the Aβ + group having significantly lower Nlgn1 band intensity than the Aβ– group (*p* = 0.019) (Fig. 5b). However, when the sample were sorted applying additionally also *t*-tau (cut-off = 460 pg/ml) and *p-*tau (cut-off = 58.3 pg/ml) criteria, the groups did not differ (Fig. 5c). Nlgn1 did not correlate with the MMSE score (rho = − 0.297) and there was no correlation between the protein and the core CSF biomarkers Aβ_42_ and *p*-tau (rho = 0.160, and 0.291, respectively), although a significant modest correlation was found with *t*-tau (rho = 0.547) and the post-synaptic biomarker neurogranin (rho = 0.512) (Fig. 5d–e).

### Confirmation of specificity of Western blot immunoreactive Nlgn1 bands by mass spectrometry

**Fig. 6:**
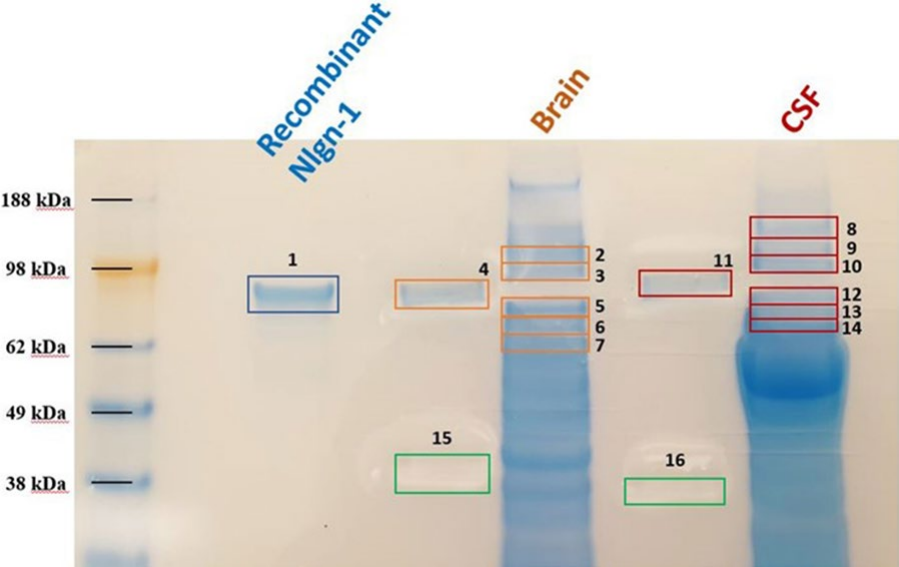
SDS-page gel (Coomassie-stained) for protein visualization and band excision for protein identification using mass spectrometry. Highlighted in the figure are the excised gel segments for the recombinant protein (1), TBS brain extract (2 to 7), and CSF sample (8 to 14). To avoid cross-contamination and protein diffusion between lanes, samples were loaded with two empty lanes in between them. Control gel segments were also cut (4, 11, 15, 16, negative controls), to further check for protein contamination between lanes. The excised gel segments were digested and protein extracted for MS analysis

The presence of Nlgn1 in CSF and brain extract was also confirmed by an antibody-independent method. To this purpose, LC–MS/MS analysis of tryptic peptides from in-gel digestion experiment was performed [60, 61]. A human brain sample (control from temporal cortex) and CSF sample were loaded on a gel together with the recombinant Nlgn1 protein. Proteins were visualized by Coomassie-staining (Fig. 6) and different segments of the gel lanes of the human brain and CSF samples, at the migration position of the recombinant Nlgn1 protein band, as well as above and below, were excised. In total, 6 gel segments for the human brain lane and 7 for the CSF sample lane were excised, respectively. The recombinant protein band was also excised and analysed as a positive control for the experiment. Excised gel segments were subjected to in-situ digestion and the tryptic peptides analysed by LC–MS for protein identification. For the recombinant protein, nine tryptic neuroligin peptides were identified, resulting in 51% sequence coverage. In brain, Nlgn1 was found in gel segment 5 and 6, and in CSF in gel segment 12 and 13, located at comparable molecular weight positions to the ones in the brain. In these bands, three unique peptides belonging to Nlgn1 were detected, together with other peptides overlapping with Nlgn2 and Nlgn3 sequences (Table 3). The proteins were not detected in the upper excised gel segments (denoted 2, 3 for brain and 8, 9, 10 for CSF) and in the control segments of the gel (denoted 4, 11, 15, 16). In spite of the presence of different neuroligin isotypes in the gel segments, it seems highly likely that the signal from the identified band in the Western blot represents Nlgn1 since the F-7 antibody was raised against a peptide specific to Nlgn1 and it was shown to not cross-react with Nlgn2 or Nlgn3 (Fig. 1).

**Table 3:** Peptides found in brain and CSF after in-gel digestion experiment

	Gel segment No.	Protein	Residues	Peptide sequence	Protein accessions
Brain sample	5	Nlgn1	267–279	GNYGLLDLIQALR	Q8N2Q7
		Nlgn1	295–320	ITVFGSGAGGScVNLLTLSHYSEGNR	Q8N2Q7
		Nlgn1-2	252–266	LGVLGFLSTGDQAAK	Q8N2Q7; Q8NFZ4
		Nlgn1-2	589–601	TGDPNQPVPQDTK	Q8N2Q7; Q8NFZ4
		Nlgn1-3	672–683	DQLYLHIGLKPR	Q8N2Q7; Q9NZ94
		Nlgn2	450–469	TLLALFTDHQWVAPAVATAK	Q8NFZ4
	6	Nlgn2	57–72	ELNNEILGPVVQFLGVPYATPPLGAR	Q8NFZ4
		Nlgn2	259–286	ITIFGSGAGAScVNLLILSHHSEGLFQK	Q8NFZ4
		Nlgn2	336–346	ELVDQDVQPAR	Q8NFZ4
		Nlgn2	315–330	VGcDREDSAEAVEcLR	Q8NFZ4
CSF sample	12	Nlgn1	267–279	GNYGLLDLIQALR	Q8N2Q7
		Nlgn1	295–320	ITVFGSGAGGScVNLLTLSHYSEGNR	Q8N2Q7
		Nlgn1	336–351	AIAQSGTALSSWAVSFQPAK	Q8N2Q7
	12–13	Nlgn1-2	252–266	LGVLGFLSTGDQAAK	Q8N2Q7; Q8NFZ4
	13	Nlgn1-2	589–601	TGDPNQPVPQDTK	Q8N2Q7; Q8NFZ4
		Nlgn1-3	672–683	DQLYLHIGLKPR	Q8N2Q7; Q9NZ94
		Nlgn2	336–346	ELVDQDVQPAR	Q8NFZ4

## Discussion

In this study, we addressed in two sets of experiments (Study 1–2) the question whether Nlgn1 protein levels are changed in TBS extracts from various brain regions from patients with neurodegenerative diseases. To our knowledge, this is the first study that looked at such changes among AD and primary tauopathy samples. For AD, we also studied whether such changes are reflected in CSF (Study 3). Nlgn1 is a trans-synaptic adhesion molecule important for synapse structure and memory formation, thus its alteration might lead to synaptic dysfunction, and neurodegenerative disease, or it could serve as a marker for such processes. Nlgn1 undergoes enzymatic cleavage, which leads to the release of a soluble extracellular fragment [32, 33], a process reminiscent of the shedding of Aβ peptides after cleavage of amyloid precursor protein (APP) by the BACE enzyme. We hypothesized that this fragment may be therefore also detectable in soluble TBS brain extract and in CSF.

The Nlgn1 levels in the brain extracts and in CSF were analysed by Western blot, with recombinant calibrator Nlgn1 fusion protein present on each blot. A band of apparently ~ 80 kDa was detected both in brain tissues and CSF samples which migrated about 5–10 kDa lower than the band of the recombinant Nlgn1 fusion protein. The Western blot reactive band appears to be a double band, which could probably reflect different Nlgn1 fragment sizes derived by enzymatic cleavages, as reported by others [32, 33]. Moreover, Nlgn1 can be spliced at two different positions, which can lead to different splice variants of the protein [62]. All of this and possible differences in the glycosylation status [33] of native Nlgn1 and the recombinant fusion protein may explain the observed different migration on the gel. However, more in depth proteomic investigations are needed to clarify these questions.

The specificity of the Nlgn1 band was initially confirmed by detection of apparently the same band with two different monoclonal antibodies to Nlgn1. Furthermore, an excised and trypsinized positive Nlgn1 band showed the presence of Nlgn proteins 1 and 2 by mass spectrometry. The F-7 antibody used in this study was raised against a Nlgn1 peptide from a region non-homologous to Nlgn2 or Nlgn3, and this could be confirmed by probing the antibody against the recombinant Nlgn1,-2 and -3 proteins, where only Nlgn1 showed a positive reactive band. Therefore, we conclude that our western blot results reflect the levels of Nlgn1. Attempts to further confirm specificity of our used mabs by immunoprecipitation (followed by Western blot and/or mass spectrometry) of brain extracts or CSF were not successful. One possible explanation is the linear epitope of the antibodies, which allows protein recognition under denaturing and reducing conditions, but not in its native form in solution, where the protein could assume different conformations.

Nlgn1 levels were assessed in both TBS extracts from human brain pathologically confirmed as brains from patients with AD, PSP, CBD, PiD and in CSF samples of AD patients. Three different human brain regions were analysed, parietal cortex, temporal cortex and frontal grey matter with 98 samples in total. Study 1 was a pilot study, where two monoclonal antibodies targeting the extracellular part of Nlgn1 were used. Both antibodies identified the same reactive band, whose intensity was reduced in the AD group.

In the second study, a cohort that we called the AD cohort was used. The Western blot analysis showed a reduction in Nlgn1 level in both sporadic and familial AD cases compared to control and PA groups. The PA term defines a group of non-demented subjects, which pathologically present with higher amyloid and tau accumulations than what it is normally expected for their age. This is not uncommon as AD pathology is also detected in non-demented elderly upon autopsy [63–65]. For this reason these subjects are classified into a disease category different from AD and could be defined as subjects having signs of pre-clinical AD [47]. This group showed levels of Nlgn1 protein similar to the control group, significantly higher than the AD groups. There were no differences between the sporadic and familial cases of AD, which might suggest that Nlgn1 levels are affected by the same pathological changes in the brain of these two groups. All the individuals in the study were then grouped according to Braak stages, Thal phases and CERAD score, independently of diagnosis. Nlgn1 levels tended to negatively associate with higher Braak stages and CERAD scores, indicating lower protein levels with increased neuritic Aβ plaques and NFTs deposition. Among the Thal phases, a significant drop of Nlgn1 levels was only seen between Thal phase 0 and Thal phase 4–5. However, it has recently been shown that Thal phases do not correlate with changes in cognitive performance and they have a low predictive value in antemortem cognition, as compared to neuritic plaques score and Braak stages [66]. This probably reflects a higher association between dense-core neuritic plaques and amyloid-related effects. Although these results might indicate an association of Nlgn1 levels to the neuropathological changes of AD, due to small sample size it is rather difficult to draw strong conclusions from this first investigation. No correlation was found with the MMSE score, suggesting that the level of the protein did not reflect decline in cognitive performance as measured by this test.

Although the focus of the study was on AD pathology, we also investigated the protein in different tauopathies, to explore Nlgn1 correlation to tau spreading. In addition, the investigation of Nlgn1 in tauopathies can provide information on the discriminatory power of Nlgn1 for different diseases, as possible diagnostic tool. AD can also be classified as a secondary tauopathy, as NFTs deposition is a hallmark of the disease and, together with Aβ plaques, it is necessary for its biological definition [17]. In AD, tau NFTs accumulation correlates better with cognitive decline than Aβ plaques and it is considered a marker of neuronal damage intensity [67, 68]. Tauopathies are classified based on the morphology of tau aggregates, their cellular location and the predominance of specific tau isoforms [69–71]. For these diseases, both *t*-tau and *p*-tau biomarkers currently available do not show good performance as they do not differ from controls [72]. For tauopathies, no other biomarkers are currently available. In the frontal grey matter, there was no significant difference between sAD and controls, likely due to high variation of the Nlgn1 levels. Across tauopathies, the protein was significantly reduced in PiD and CBD compared to controls, but not in PSP. However, the same trend was seen also in PSP. Nlgn1 levels could not discriminate between the different tauopathies. Comparing all the different brain regions, namely parietal cortex, temporal cortex and frontal grey matter, there were no significant differences in the Nlgn1 protein levels (data not shown).

In Study 4, we aimed to investigate the existence of Nlgn1 in CSF. CSF is in close proximity to the CNS [73] and is therefore regarded as a biofluid holding great potential for biomarkers for biochemical changes in the brain. Western blot analysis of a cohort of clinically classified AD and control cases showed a band of the same apparent molecular weight as detected in soluble brain homogenate fractions. The analysis showed a significant reduction on the band intensity in the AD group. Further, the groups were dichotomized in Aβ + and Aβ-, based on the ratio Aβ_42/40_. The use of the ratio has been shown to increase the diagnostic accuracy when compared to the use of Aβ_42_ alone [16] and to compensate for between-individual variations in total Aβ production [74], as for example seen in the PA group. In the dichotomized groups based on the Aβ ratio, the significant difference was diminished and it was abolished when the other biomarkers cut-offs, *t*-tau and *p*-tau, were applied. Nlgn1 values correlated with *t*-tau, but not with *p*-tau. These results suggest that Nlgn1 does not represent a promising marker for AD pathological changes in CSF. A good correlation was seen with the postsynaptic protein neurogranin, which may suggest that Nlgn1 alteration is indicative of general synaptic dysfunction and neuronal damage.

In conclusion, many studies have described the possible implication of Nlgn1 dysfunction in cognitive disorders [37, 39, 41, 75–77], which make the protein an interesting synaptic biomarker candidate to study. Nlgn1 has been shown to be decreased in plasma neuronal exosomes [78], but no other investigations have been performed on Nlgn1. Our results show that the protein is reduced in different brain regions of AD patients, in accordance with recent investigations [45]. The protein is also detectable in CSF, where it is decreased in the AD group, however showing considerable overlap with the control group, thus probably not representing a good synaptic biomarker for AD pathology. However, Nlgn1 levels showed the greatest reduction in frontal grey matter of different tauopathies, which raises the interest for further investigations in the CSF of these patients and in possible roles of Nlgn1 shedding in primary tau pathologies. In spite of the well characterized brain cohorts of this study, this investigation has several limitations which should be mentioned, such as the small sample size, and the technical limitations of protein quantitation by western blot. In addition, the limited size resolution of western blots did not allow us to quantify the two Nlgn1 bands separately. Therefore, future work could investigate if the protein is present in CSF as fragments of various lengths, isoforms, or with different posttranslational modifications, such as glycosylations. It would also be of interest to evaluate if the other neuroligin family members are altered under pathological conditions, as proteomic studies have shown elevated levels of Nlgn2 peptides in the CSF of prodromal AD and AD dementia patients [79]. Ultimately, investigating the shedding of the protein could add important information to the understanding of the mechanisms regulating synapses, both physiologically and under pathological conditions.

## Supplementary Information

**Additional file 1: Table S1.** Full case demographics of the AD cohort from temporal cortex. The ABC score is a composite of three different assessments and it incorporates (A) Thal phases of amyloid deposition, (B) Braak stage of NFTs and (C) score of amyloid neuritic plaques (CERAD). PM= post-mortem delay. NFTs=neurofibrillary tangles. CERAD= consortium to establish a registry for Alzheimer´s disease. CAA= cerebral amyloid angiopathy.

**Additional file 2: Table S2.** Full case demographics of the tauopathies cohort from frontal grey matter. The ABC score is a composite of three different assessments and it incorporates (A) Thal phases of amyloid deposition, (B) Braak stage of NFTs and (C) score of amyloid neuritic plaques (CERAD). AAO=age at onset, AAD=age at death, PM delay= post-mortem delay, CERAD= consortium to establish a registry for Alzheimer´s disease, CAA= cerebral amyloid angiopathy, na= non-available.

## Data Availability

The datasets during and/or analysed during the current study are available from the corresponding author on reasonable request.

## Abbreviations

Nlgn1: Neuroligin-1
CNS: Central nervous system
LTP: Long-term potentiation
AD: Alzheimer´s disease
CSF: Cerebrospinal fluid
APP: Amyloid precursor protein
Aβ: Amyloid-beta
Aβo: Aβ oligomers
WB: Western blot
NFTs: Neurofibrillary tangles
CBD: Corticobasal degeneration
PSP: Progressive supranuclear palsy
PiD: Pick’s disease
PA: Pathological aging
Ctrl: Control
MMSE: Mini-Mental State Examination
LDS: Lithium dodecyl sulphate
TBS: Tris-buffered saline
PBST: Phosphate-buffered saline with tween
DTT: Dithiothreitol
LC–MS: Liquid chromatography-mass spectrometry
sAD: Sporadic Alzheimer´s disease
fAD: Familial cases/early onset Alzheimer´s disease
