# Supplementary material for: Neuroligin-1 in brain and CSF of neurodegenerative disorders: investigation for synaptic biomarkers
Source: Acta Neuropathol Commun. 2021 Feb 1;9:19. doi: 10.1186/s40478-021-01119-4 (PMC7852195; doi:10.1186/s40478-021-01119-4)
Supplement: Supplementary file 1 — Additional file 1: Table S1. Full case demographics of the AD cohort from temporal cortex. The ABC score is a composite of three different assessments and it incorporates (A) Thal phases of amyloid deposition, (B) Braak stage of NFTs and (C) score of amyloid neuritic plaques (CERAD). PM= post-mortem delay. NFTs=neurofibrillary tangles. CERAD= consortium to establish a registry for Alzheimer´s disease. CAA= cerebral amyloid angiopathy. [file 40478_2021_1119_MOESM1_ESM.docx]

| **Case Number** | **Diagnosis** | **Gender** | **Age at death** | **PM delay**  **(h)** | **Brain Weight**  **(g)** | **ApoE**  **genotype** | **Mutation** | **Braak staging (NFTs)** | **Thal Phase** | **CERAD score** | **ABC score** | **CAA** |
| --- | --- | --- | --- | --- | --- | --- | --- | --- | --- | --- | --- | --- |
| 1 | normal controls | M | 38 | 80.35 | 1581 | ε3/ε4 | na | 0 | 1 | 0 | A1B0C0 | 0 |
| 2 | normal controls | M | 77 | 40.1 | 1327 | ε2/ε2 | na | 0 | 0 | 0 | A0B0C0 | 2 |
| 3 | normal controls | F | 86 | 40.2 | 1238 | ε3/ε3 | na | 0 | 0 | 0 | A0B0C0 | 0 |
| 4 | normal controls | M | 95 | 89.4 | 1346 | ε2/ε3 | na | I | 2 | 1 | A1B1C1 | 1 |
| 5 | normal controls | F | 84 | 79.1 | 1468 | ε3/ε3 | na | 0 | 0 | 0 | A0B0C0 | 0 |
| 6 | normal controls | F | 68 | 45.05 | 1330 | ε2/ε3 | na | 0 | 0 | 0 | A0B0C0 | 0 |
| 7 | normal controls | M | 71 | 38.5 | 1480 | ε3/ε3 | na | I | 0 | 0 | A0B1C0 | 0 |
| 8 | sAD | M | 72 | 42.15 | 1217 | ε3/ε4 | na | VI | 5 | 3 | A3B3C3 | 1 |
| 9 | sAD | F | 86 | 90.2 | 1065 | ε3/ε4 | na | VI | 5 | 3 | A3B3C3 | 3 |
| 10 | sAD | M | 69 | 64.55 | 1040 | ε2/ε3 | na | V | 5 | 3 | A3B3C3 | 1 |
| 11 | sAD | M | 81 | 78.15 | 1116 | ε3/ε3 | na | VI | 5 | 2 | A3B3C3 | 2 |
| 12 | sAD | F | 62 | 62.55 | 978 | ε3/ε4 | na | VI | 5 | 3 | A3B3C3 | 3 |
| 13 | sAD | M | 65 | 96.3 | 1270 | ε3/ε3 | na | VI | 5 | 3 | A3B3C3 | 2 |
| 14 | sAD | M | 68 | 52.05 | 1154 | ε3/ε4 | na | VI | 5 | 3 | A3B3C3 | 1 |
| 15 | sAD | M | 80 | 62.1 | 1309 | ε3/ε4 | na | VI | 5 | 3 | A3B3C3 | 1 |
| 16 | sAD | F | 66 | 92.47 | 906 | ε3/ε3 | na | VI | 5 | 3 | A3B3C3 | 2 |
| 17 | fAD | F | 65 | 31.55 | 762 | ε3/ε4 | PS1 R278I | VI | 5 | 3 | A3B3C3 | 3 |
| 18 | fAD | F | 52 | 32.3 | 763 | ε4/ε4 | PS1 Intron4 | VI | 5 | 3 | A3B3C3 | 3 |
| 19 | fAD | F | 37 | 24.15 | 1182 | ε3/ε3 | PS1 E120K | VI | 5 | 3 | A3B3C3 | 3 |
| 20 | fAD | F | 56 | 16.25 | 997 | ε3/ε3 | APP V717I | VI | 5 | 3 | A3B3C3 | 3 |
| 21 | fAD | M | 66 | 68.05 | 1437 | ε3/ε3 | APP V717I | VI | 5 | 3 | A3B3C3 | 1 |
| 22 | fAD | M | 47 | 43.5 | 1225 | ε3/ε3 | PS1 A434T & T291A | VI | 5 | 3 | A3B3C3 | 3 |
| 23 | fAD | F | 41 | 64.15 | 1108 | ε3/ε3 | PS1 Intron4 | VI | 5 | 3 | A3B3C3 | 2 |
| 24 | fAD | F | 59 | 26.15 | 1038 | ε4/ε4 | PS1 I202F | VI | 5 | 3 | A3B3C3 | 3 |
| 25 | fAD | M | 51 | 43.1 | 1415 | ε3/ε3 | PS1 Intron4 | VI | 5 | 3 | A3B3C3 | 3 |
| 26 | Path. ageing | F | 94 | 109 | 1541 | ε3/ε4 | na | III | 3 | 1 | A2B2C1 | 0 |
| 27 | Path. ageing | M | 83 | 105.28 | 1244 | ε3/ε3 | na | IV | 3 | 2 | A2B2C2 | 2 |
| 28 | Path. ageing | F | 86 | 119.05 | 1230 | ε3/ε4 | na | I | 4 | 1 | A3B1C1 | 0 |
| 29 | Path. ageing | F | 82 | 99.2 | 1263 | ε3/ε3 | na | II | 5 | 1 | A3B1C1 | 0 |
| 30 | Path. ageing | F | 71 | 76.1 | 1214 | ε3/ε3 | na | III | 2 | 0 | A1B2C0 | 2 |
| 31 | Path. ageing | F | 91 | 19.4 | 1154 | ε3/ε3 | na | IV | 5 | 2 | A3B2C2 | 2 |
| 32 | Path. ageing | F | 91 | 69.2 | 1311 | ε3/ε3 | na | IV | 4 | 2 | A3B2C2 | 1 |
| 33 | Path. ageing | M | 88 | 97.3 | 1629 | ε3/ε3 | na | II | 3 | 1 | A2B1C1 | 1 |
| 34 | Path. ageing | F | 95 | 39 | 1169 | ε3/ε3 | na | IV | 4 | 2 | A3B2C2 | 0 |
| 35 | Path. ageing | F | 89 | 38.29 | 1448 | ε3/ε3 | na | III | 3 | 1 | A2B2C1 | 0 |
| 36 | Path. ageing | M | 88 | 32.3 | 1306 | ε3/ε4 | na | III | 5 | 2 | A3B2C2 | 2 |
| 37 | Path. ageing | M | 85 | 77.5 | 1393 | ε2/ε3 | na | III | 1 | 1 | A1B2C1 | 2 |
| 38 | Path. ageing | F | 88 | 99 | 1160 | ε3/ε3 | na | IV | 2 | 0 | A1B2C0 | 0 |
| 39 | Path. ageing | F | 82 | 91.2 | 1262 | ε3/ε4 | na | II | 3 | 1 | A2B1C1 | 2 |
| 40 | Path. ageing | F | 82 | 72 | 1228 | ε3/ε3 | na | III | 1 | 1 | A1B2C1 | 3 |

***Supplementary table 1***: full case demographics of the AD cohort from temporal cortex. The ABC score is a composite of three different assessments and it incorporates (A) Thal phases of amyloid deposition, (B) Braak stage of NFTs and (C) score of amyloid neuritic plaques (CERAD). PM= post-mortem delay. NFTs=neurofibrillary tangles. CERAD= consortium to establish a registry for Alzheimer´s disease. CAA= cerebral amyloid angiopathy.
