# Supplementary material for: Neuroligin-1 in brain and CSF of neurodegenerative disorders: investigation for synaptic biomarkers
Source: Acta Neuropathol Commun. 2021 Feb 1;9:19. doi: 10.1186/s40478-021-01119-4 (PMC7852195; doi:10.1186/s40478-021-01119-4)
Supplement: Supplementary file 2 — Additional file 2: Table S2. Full case demographics of the tauopathies cohort from frontal grey matter. The ABC score is a composite of three different assessments and it incorporates (A) Thal phases of amyloid deposition, (B) Braak stage of NFTs and (C) score of amyloid neuritic plaques (CERAD). AAO=age at onset, AAD=age at death, PM delay= post-mortem delay, CERAD= consortium to establish a registry for Alzheimer´s disease, CAA= cerebral amyloid angiopathy, na= non-available. [file 40478_2021_1119_MOESM2_ESM.docx]

| **Case Number** | **Diagnosis** | **Gender** | **AAO**  **(years)** | **AAD**  **(years)** | **Duration** | **PM delay**  **(h)** | **Brain Weight**  **(g)** | **ApoE**  **genotype** | **Braak staging**  **(NFTs)** | **Thal Phase** | **CERAD**  **score** | **ABC**  **score** | **CAA** |
| --- | --- | --- | --- | --- | --- | --- | --- | --- | --- | --- | --- | --- | --- |
| 41 | AD | M | 54 | 65 | 11 | 34.25 | 1089 | ε4/ε4 | VI | 5 | 3 | A3B3C3 | 3 |
| 42 | AD | M | 48 | 63 | 15 | 31.42 | 1042 | ε3/ε3 | VI | 5 | 3 | A3B3C3 | 3 |
| 43 | AD | M | 44 | 59 | 15 | 90.45 | 1338 | na | VI | 5 | 3 | A3B3C3 | 3 |
| 44 | AD | F | 69 | 76 | 7 | 42 | 1236 | na | VI | 5 | 2 | A3B3C2 | 0 |
| 45 | AD | M | 71 | 86 | 15 | 95.10 | 1203 | ε3/ε3 | V | 5 | 3 | A3B3C3 | 3 |
| 46 | AD | M | 54 | 67 | 13 | 32.15 | 1458 | ε4/ε4 | VI | 5 | 3 | A3B3C3 | 3 |
| 47 | AD | F | 49 | 69 | 20 | 40.10 | 986 | ε4/ε4 | VI | 5 | 2 | A3B3C2 | 3 |
| 48 | AD | M | 52 | 71 | 19 | 45.35 | 1097 | ε3/ε3 | VI | 5 | 3 | A3B3C3 | 3 |
| 49 | AD | F | 58 | 62 | 4 | 92.20 | 1234 | ε3/ε4 | VI | 5 | 2 | A3B3C2 | 1 |
| 50 | AD | F | 57 | 76 | 19 | 57.50 | 1303 | ε3/ε4 | VI | 5 | 2 | A3B3C2 | 2 |
| 51 | Control | M | na | 101 | na | 60.25 | 1450 | ε2/ε3 | I | 0 | 0 | A0B1C0 | 1 |
| 52 | Control | M | na | 38 | na | 80.35 | 1581 | ε3/ε4 | 0 | 0 | 0 | A0B0C0 | 0 |
| 53 | Control | F | na | 86 | na | 119.05 | 1230 | ε3/ε4 | I | 4 | 1 | A3B1C1 | 0 |
| 54 | Control | F | na | 87 | na | 51.40 | 1114 | ε3/ε3 | I | 1 | 0 | A1B1C1 | 0 |
| 55 | Control | F | na | 86 | na | 40.20 | 1238 | ε3/ε3 | 0 | 0 | 0 | A0B0C0 | 0 |
| 56 | Control | F | na | 78 | na | 29.30 | 1225 | ε2/ε2 | I | 1 | 0 | A0B1C0 | 0 |
| 57 | Control | F | na | 68 | na | 45.05 | 1330 | ε2/ε3 | 0 | 0 | 0 | A0B0C0 | 0 |
| 58 | Control | M | na | 69 | na | 171 | 1435 | ε3/ε3 | I | 3 | 1 | A2B1C1 | 1 |
| 59 | Control | F | na | 79 | na | 88.50 | 1288 | ε3/ε3 | I | 2 | 1 | A2B1C1 | 0 |
| 60 | Control | M | na | 95 | na | 89.40 | 1346 | ε2/ε3 | I | 2 | 1 | A2B1C1 | 0 |
| 61 | PSP | M | 73 | 84 | 11 | 66.34 | 1271 | na | na | na | na | na | na |
| 62 | PSP | F | 66 | 79 | 13 | 73.55 | 1141 | na | na | na | na | na | na |
| 63 | PSP | M | 57 | 62 | 5 | 72.20 | 1369 | na | na | na | na | na | na |
| 64 | PSP | M | 76 | 84 | 8 | 50 | 1370 | na | na | na | na | na | na |
| 65 | PSP | F | 75 | 84 | 9 | 70 | 1095 | na | na | na | na | na | na |
| 66 | PSP | M | 71 | 83 | 12 | 32.35 | 1137 | na | na | na | na | na | na |
| 67 | PSP | F | 60 | 68 | 8 | 36.50 | 1177 | na | na | na | na | na | na |
| 68 | PSP | F | 84 | 92 | 8 | 43.10 | 1118 | na | na | na | na | na | na |
| 69 | PSP | F | 67 | 77 | 10 | 30.42 | 1095 | na | na | na | na | na | na |
| 70 | PSP | M | 78 | 88 | 10 | 48.45 | 1307 | na | na | na | na | na | na |
| 71 | PSP | M | 63 | 71 | 8 | 4.35 | 1179 | na | na | na | na | na | na |
| 72 | CBD | M | 63 | 69 | 6 | 81.36 | 1291 | na | na | na | na | na | na |
| 73 | CBD | M | 56 | 62 | 6 | 45.15 | 1173 | na | na | na | na | na | na |
| 74 | CBD | F | 58 | 69 | 11 | 103.15 | 917 | na | na | na | na | na | na |
| 75 | CBD | M | 54 | 61 | 7 | 102.30 | 1389 | na | na | na | na | na | na |
| 76 | CBD | M | 58 | 65 | 7 | 48.04 | 1232 | na | na | na | na | na | na |
| 77 | CBD | M | 69 | 77 | 8 | 37 | 1113 | na | na | na | na | na | na |
| 78 | CBD | F | 62 | 68 | 6 | 98.25 | 1172 | na | na | na | na | na | na |
| 79 | CBD | M | 67 | 73 | 6 | 54.25 | 1154 | na | na | na | na | na | na |
| 80 | CBD | M | 57 | 72 | 15 | 84.10 | 1378 | na | na | na | na | na | na |
| 81 | CBD | F | 66 | 70 | 4 | 75 | 1050 | na | na | na | na | na | na |
| 82 | PiD | F | 60 | 71 | 11 | 87.15 | 849 | na | na | na | na | na | na |
| 83 | PiD | M | 52 | 67 | 15 | 30.30 | 982 | na | na | na | na | na | na |
| 85 | PiD | M | 57 | 62 | 5 | 24 | 1166 | na | na | na | na | na | na |
| 86 | PiD | M | 70 | 78 | 8 | 74.45 | 1280 | na | na | na | na | na | na |
| 87 | PiD | M | 53 | 67 | 14 | 73.05 | 1000 | na | na | na | na | na | na |
| 88 | PiD | M | 51 | 68 | 17 | 79.50 | 854 | na | na | na | na | na | na |
| 89 | PiD | M | 63 | 75 | 12 | 46.30 | 933 | na | na | na | na | na | na |
| 90 | PiD | M | 60 | 68 | 8 | 94.45 | 1209 | na | na | na | na | na | na |
| 91 | PiD | M | 55 | 72 | 17 | 97.35 | 805 | na | na | na | na | na | na |

***Supplementary table 2***: full case demographics of the tauopathies cohort from frontal grey matter. The ABC score is a composite of three different assessments and it incorporates (A) Thal phases of amyloid deposition, (B) Braak stage of NFTs and (C) score of amyloid neuritic plaques (CERAD). AAO=age at onset, AAD=age at death, PM delay= post-mortem delay, CERAD= consortium to establish a registry for Alzheimer´s disease, CAA= cerebral amyloid angiopathy, na= non-available.
